# Effects of Collagen Grafting on Cell Behaviors in BCP Scaffold with Interconnected Pore Structure

**DOI:** 10.1186/s40824-016-0049-3

**Published:** 2016-01-15

**Authors:** Dong-Jun Yang, Jae-Hui Jeon, Sun-Young Lee, Hyun-Wook An, Keun Oh Park, Kwang-Bum Park, Sukyoung Kim

**Affiliations:** Department of Institute of Science & Technology, Megagen Implant, Jain-myeon, Gyeongsan, Gyeongbuk 712-852 Korea; School of Materials Science & Engineering, Yeungnam University, Gyeongsan, Gyeongbuk 712-749 Korea

**Keywords:** Hydroxyapatite (HA), Tricalcium phosphate (TCP), Biphasic calcium phosphate (BCP), Collagen

## Abstract

**Background:**

This study was to investigate the effect of collagen grafted porous biphasic calcium phosphate (BCP) on cell attachment, proliferation, and differentiation. Porous BCP scaffolds with interconnected micropore structure were prepared with were prepared and then grafted with a collagen type I. The hydroxyapatite (HA) and β-tricalcium phosphate (TCP) ratio of the TCP scaffolds was about 60/40 and the collagen was crosslinked on the TCP scaffold surface (collagen-TCP).

**Results:**

The sintered BCP scaffolds showed fully interconnected micropore structures with submicron-sized grains. The collagen crosslinking in the scaffolds was conducted using the the N-(3-Dimethylaminopropyl)-N′-ethylcarbodiimide hydrochloride and N-hydroxysuccinimide (NHS) crosslinking method. The cell proliferation of collagen-BCP scaffolds showed a similar result to that of the BCP scaffolds. However, osteoblastic differentiation and cell attachment increased in the collagen-BCP scaffolds.

**Conclusions:**

Collagen-BCP scaffold improved the cell attachment ability in early phase and osteoblastic differentiation.

## Background

Autograft, xenograft, and synthetic grafting bone substitutes with diverse chemical compositions are widely used as an alternative to autogenous grafting material to repair osseous defects in dentistry [1–5]. The calcium phosphate such as hydroxyapatite (HA), β-tricalcium phosphate (β-TCP) and biphasic calcium phosphate (BCP) are commonly used as a bone substitute due to their excellent biocompatibility. The most synthetic BCP bone substitute consists of a mixture of HA and β-TCP with various ratio. HA and β-TCP are very different in terms of the solubility or dissolution rate, which reflects their bioreactivity. β-TCP resorbs more quickly than HA [6]. Therefore, the bioreactivity of BCP can be controlled by changing the ratio of HA and β-TCP [7].

The structural characteristics and chemical composition of BCP scaffolds play a critical role in osteoconductivity of bone substitute. In structural aspects, the pore size at both macro- and micro-levels, porosity and the interconnection of microspores are important factors for bone healing [8–13]. In recent study, donut shape BCP bone substitutes made of a central macro-pore (about 300 ~ 400 μm) and micro-pores (about 20–60 μm) showed greater new bone formation when compared with similar BCP composition with micro-pores [14]. In general, BCP with various HA/TCP ratio shows greater new bone formation when compared with HA and β-TCP.

Collagen which is one of extracellular components of bone tissue promotes osteogenic differentiation of osteoblast and mesenchymal stem cells *in vitro* [15–18]. It is known that HA functionalized with collagen I affects the cell adhesion and mineralization of mesenchymal stem cells [19]. And collagen-TCP porous ceramics are used in human extraction socket healing and forms sufficient amounts of vital bone [20].

This study aimed to investigate the cell behaviors such as cell attachment, proliferation, and differentiation in porous BCP ceramics. Especially, the effect of collagen crosslinked on BCP ceramic surface was examined. In order to compare the cell behaviors between pure BCP and collagen grafted BCP ceramics (collagen-BCP) with interconnected micropore structures, collagen-BCP samples were prepared by crosslinking the N-(3-Dimethylaminopropyl)-N′-ethylcarbodiimide hydrochloride and N-hydroxysuccinimide (NHS) on pure BCP ceramics. It is known that the compound of EDC and NHS is a coupling agent and efficient and non-toxic crosslinking material [21–23].

## Methods

### Preparation of BCP scaffolds

BCP powder was synthesized by a precipitation method using 14.17 g of Ca (NO3)2·4H2O (Duksan Pure Chemicals; Gyunggi-do, Korea) and 5.11 g of (NH_4_) _2_·HPO_4_ (Duksan Pure Chemicals; Gyunggi-do, Korea). First, Ca (NO_3_) _2_·4H_2_O and (NH_4_) _2_·HPO_4_ were dissolved in distilled water and (NH_4_) _2_·HPO_4_ solution was added drop by drop to the Ca (NO_3_) _2_·4H_2_O solution. The pH of the solution was adjusted to 8.5 with ammonium hydroxide (Duksan) after dissolved completely at 80 °C. And the solution was stirred for 1 h, washed with distilled water to remove ammonium hydroxide and filtered with 0.2 μm membrane filter. The filter cake was crushed and dried in a drying oven for 12 h. The as-dried powder was then calcined at 900 °C for 1 h. The donut shape porous BCP samples were produced with the calcined powder.

### Collagen crosslinking

The collagen on the BCP scaffold surface was chemically crosslinked. First, 5 % collagen was dispersed in 1 % acetic acid at 0 ~ −5 °C for 6 ~ 12 h. A mixture of 0.05 g N-(3-dimethylaminopropyl)-N’-ethylcarbodiimide hydrochloride (EDC, Sigma-Aldrich Canada, Ltd; Oakville, Canada) and 0.05 g N-hydroxysuccinimide (NHS, Sigma-Aldrich Canada, Ltd; Oakville, Canada) was prepared in distilled water as described previously [21–23]. Carbodiimide crosslinking in collagen solution by using EDC and NHS was performed by reacting the two solutions at 0 ~ −5 °C for 24 h in ice bath. In order to crosslink the collagen on BCP surface, the BCP scaffolds were immersed in 10 % 3-aminopropyltriethoxysilane (3-APTES) at 95 °C for 2 h, washed three times with distilled water and dried in a drying oven. The crosslinking of amino group on the scaffold surface was performed via the 3-APTES terminal amino group. The 3-APTES treated BCP scaffolds with amino groups reacted with the prepared collagen solution at room temperature for 6 h. Collagen treated BCP samples (collagen-TCP) were washed three times with distilled water and dried.

### X-ray diffraction (XRD)

Both BCP scaffolds before and after collagen crosslinking (TCP and collagen-TCP) were analyzed to examine the crystalline phases (HA and TCP) with X-ray diffractometer (DMAX-2500, RIGAKU, Japan). The diffractometer was operated at 40 kV and 30 mA employing a step size of 1°/min.

### Scanning electron microscopy (SEM)

Surface morphology of both scaffolds was observed using scanning electron microscope (SEM) equipped with energy dispersive X-spectroscope (EDS) (Hitachi S-4200, Tokyo, Japan). Accelerating voltage was set as 15 kV.

### X-ray photoelectron spectroscopy

In order to confirm the collagen crosslinked on BCP surface, X-ray photoelectron spectroscopy (XPS, Quantera SXM, ULVAC-PHI, Japan) was used.

### Coomassie brilliant blue staining

Scaffolds were stained in 0.1 % Coomassie brilliant blue R250 for 20 min and destined in 45 % methanol and 10 % glacial acetic acid until the background of the gel was removed.

### Cell attachment

The MC3T3-E1 cells (2 × 10^4^ cells), a mouse calvaria-derived osteoblast-like cell line, and implants in α-modified Eagle’s medium (α-MEM) were repeatedly rotated by using a rotation plate (2 rpm) in a flat-bottom tube at 37 °C for 3 h [24]. The cells on three samples (control HA, pure BCP and collagen-BCP) were incubated in a 5 % CO_2_ incubator at 37 °C for 3 h. After incubation, the scaffolds were washed twice with phosphate buffered saline (pH 7.4). Fixation was carried out for 30 min in 2 % glutaraldehyde. The scaffold samples were then washed twice with 0.1 M sodium cacodylate buffer (pH 7.4), dehydrated sequentially in 25 %, 50 %, 75 %, 95 %, and 100 % ethanol, for 5 min each, and dried with tetramethylsilane. The scaffold specimens were coated with gold, examined, and photographed using a SEM equipped with an EDS (SEM/EDS, S-4800, Hitachi, Tokyo, Japan).

### Cell proliferation

The MC3T3-E1 cells were seeded into 24-well plates at a density of 2 × 10^4^ cells per well. After 24 h, control, pure BCP and collagen-BCP scaffolds were added into each well. The cells on three samples were incubated in a 5 % CO_2_ incubator at 37 °C for 1, 4 and 7 days. MTT (3-(4,5-dimethylthiazol-2yl)-2,5-diphenyl tetrazolium bromide) assay was performed for the cell proliferation at 1, 4, and 7 days. 0.5 mg/ml of MTT solution was added to each well. After 3 h, the MTT solution was aspirated and the dimethylsulfoxide was added to solubilize the formed formazan. The optical density was measured at a wavelength of 570 nm using an ELISA reader (PowerWave XS, Bio-Tek, Winooski, USA). Cell counting was performed for the cell quantification for 3, 5, and 7 days. The cells were detached from culture plate, and washed with PBS. The cells were counted by using a haemocyotometer.

### Alkaline phosphate (ALP) staining

The MC3T3-E1 cells were seeded into 24-well plates at a density of 2 × 10^4^ cells per well. After 24 h, the media was changed with osteogenic medium and then pure BCP and collagen-BCP scaffolds were added into each well. The cells were incubated at 37 °C in a humidified atmosphere of 5 % CO_2_ for 7 days. The cells were washed PBS and the ALP staining was performed using alkaline phosphatase (ALP) Kit (SIGMA-ALDRICH, INC; St. Louis, MO, USA).

### Statistical analysis

Statistical analysis was performed by a using SPSS 11.0 statistical system (SPSS Inc., Chicago, IL, USA). The paired Student *t*-test was performed to compare the significance of the differences in cell proliferation. Values of *p* were statistically significant at < 0.05.

## Results and discussion

### Characterization of BCP and collagen-BCP Scaffolds

**Fig. 1:**
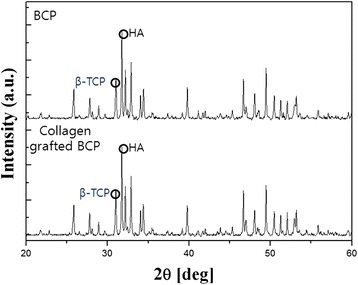
X-ray diffraction of BCP scaffolds and collagen/ BCP scaffolds

The crystallinity and phase composition in pure BCP and collagen-BCP scaffolds were investigated by using XRD. X-ray diffraction patterns of pure BCP and collagen-BCP scaffolds are consisted of two phases (HA and β-TCP) and is shown in Fig. 1. The ratio of HA and β-TCP phases calculated by Rietveld method in both BCP scaffolds was 60/40 and did not show any change of crystallinity.

**Fig. 2:**
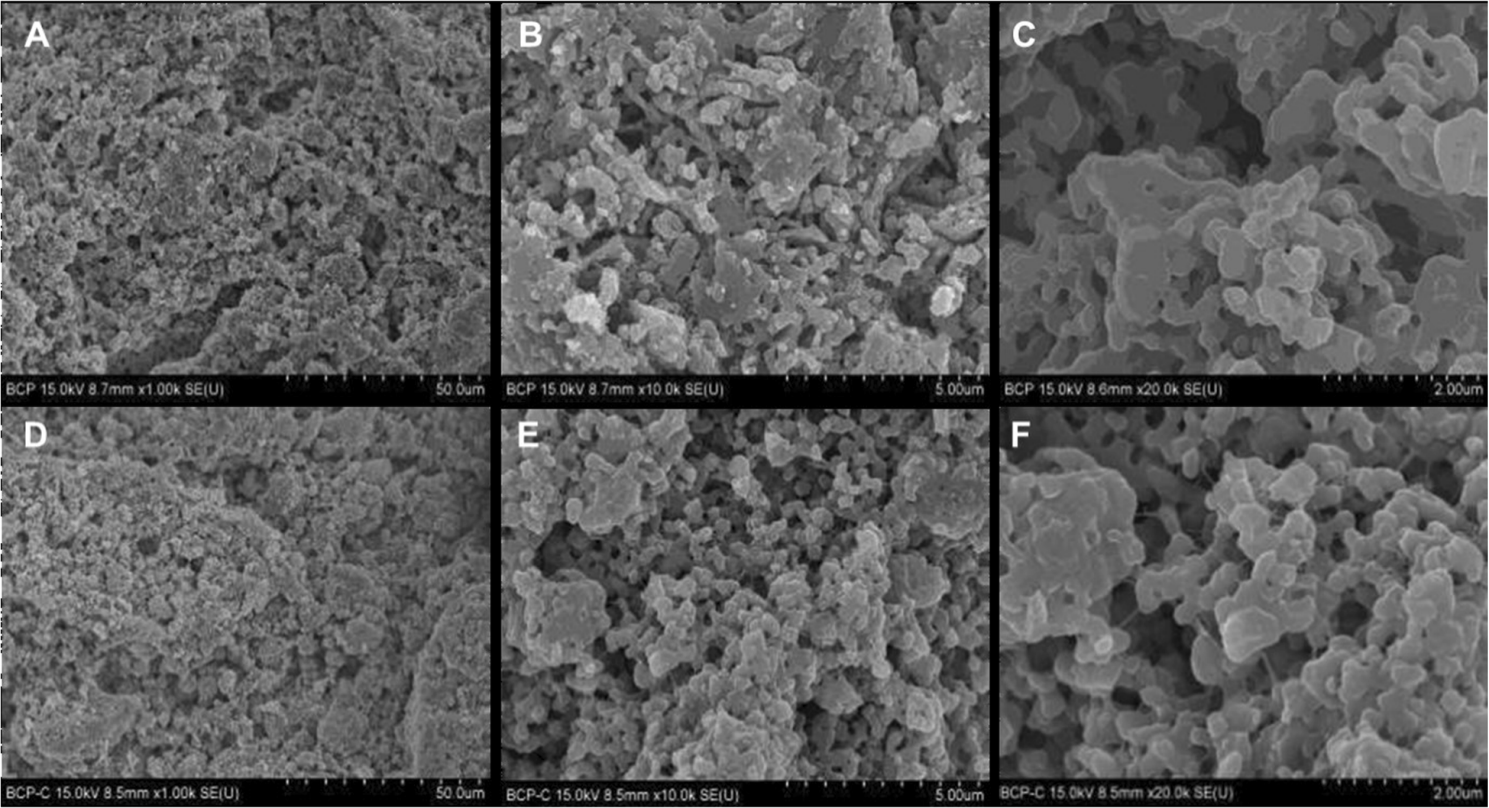
Scanning electron microscope image of BCP scaffolds and collagen/ BCP scaffolds. SEM images of BCP scaffolds (**a**, **b**, **c**) and collagen/ BCP scaffolds (**d**, **e**, **f**) at magnifications of × 1000 (**a**, **d**), ×10000 (**b**, **e**) and × 20000 (**c**, **f**)

The surface topography in both BCP and collagen-BCP scaffolds was observed by using SEM (Fig. 2). SEM images show submicron-sized grains with interconnected micropore structures in the BCP and collagen-BCP scaffold. The collagen-BCP scaffold had a similar surface morphology to the BCP scaffold at low magnification images but showed the collagen on the surface at higher magnifications.

**Fig. 3:**
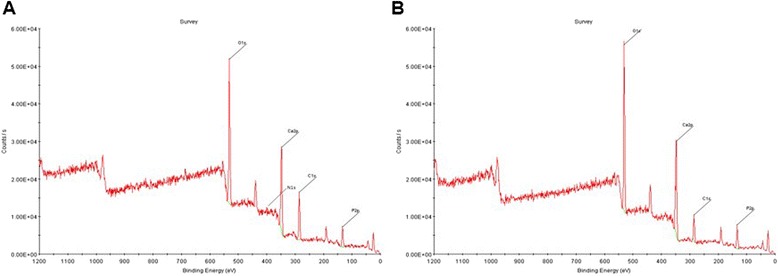
X-ray photoelectron spectroscopy spectra of BCP scaffolds and collagen/ BCP scaffolds. XPS of BCP scaffolds (**a**) and collagen/ BCP scaffolds (**b**)

In order to confirm the collagen grafting, XPS analysis was conducted (Fig. 3). The N_1s_ (nitrogen peak) on the collagen-BCP scaffold was observed in the XPS pattern (Fig. 3-b). An observation of N_1s_ signal in the XPS pattern means the presence of amino group of collagen and the crosslinking of collagen on BCP scaffolds.

**Fig. 4:**
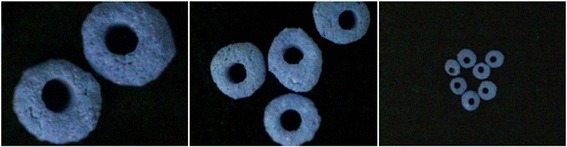
Coomassie brilliant blue staining of collagen/ BCP scaffolds

The presence of collagen on collagen-BCP scaffold was also observed by using Coomassie brilliant blue staining (Fig. 4). Coomassie brilliant blue staining is generally used for detection of protein on sodium dodecyl sulfate polyacrylamide gel electrophoresis (SDS-PAGE) and widely used in a various area. The color of BCP scaffolds is purple if the collagen is present on the surface. The Coomassie brilliant blue is binding with collagen. This method has an advantage that the presence of collagen on specimen can distinguish with the naked eye without using equipment such as SEM and XPS etc. The purple color on collagen-BCP scaffold was observed on all surfaces.

Therefore, it is demonstrated that the collagen was crosslinked efficiently on BCP scaffolds using EDC/ NHS method and the crosslinking of collagen did not affect overall structure of scaffolds.

### Behaviors of osteoblastic cells on collagen-BCP Scaffold

**Fig. 5:**
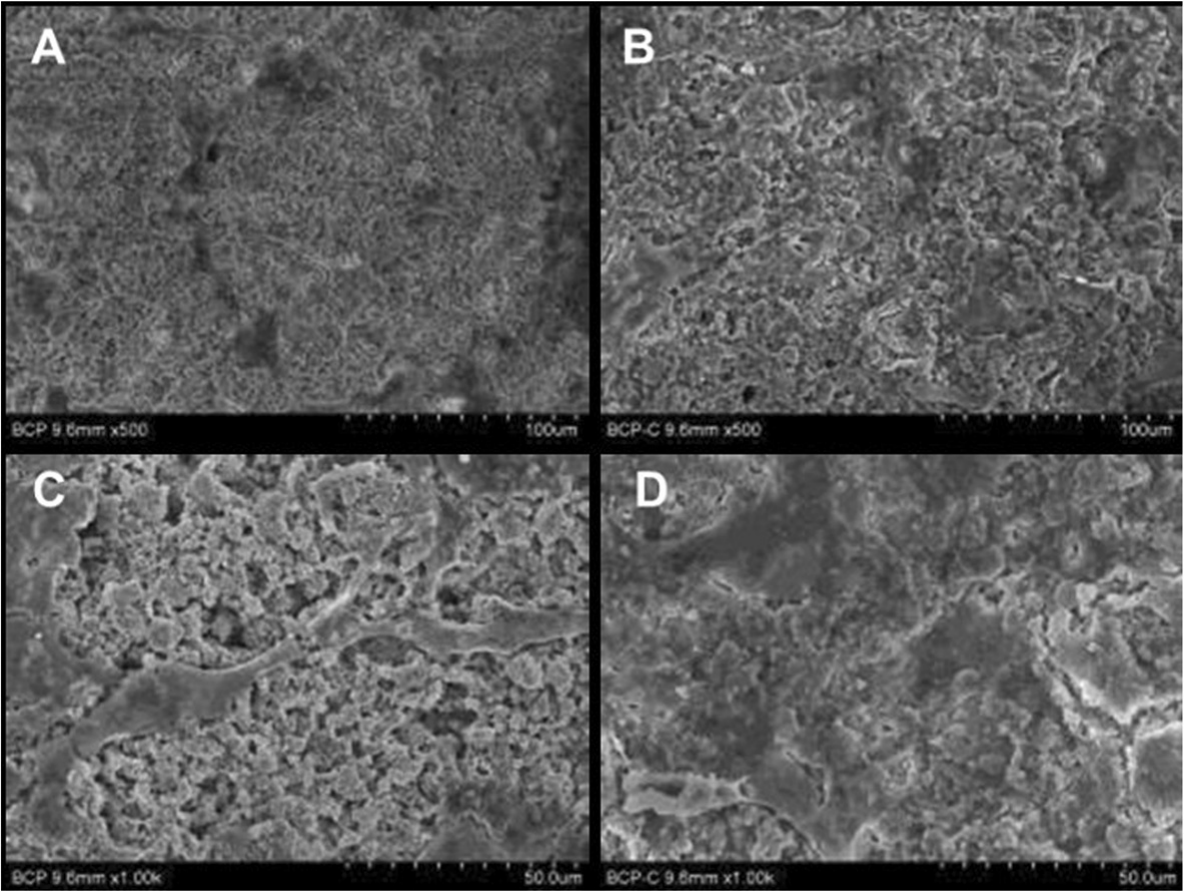
Cell morphology of MC3T3-E1 on BCP scaffolds and collagen/BCP scaffolds. SEM images of MC3T3-E1 cultured on BCP scaffolds (**a**, **c**) and collagen-BCP scaffolds (**b**, **d**) for 24 h at magnifications of × 500 (**a**, **b**) and × 10000 (**c**, **d**)

To evaluate the effects of collagen on cell attachment, MC3T3-E1 cells were cultured on the BCP scaffold and the collagen-BCP scaffold for 24 h, and then the cell morphology was observed by using SEM. The cells on the collagen-BCP scaffold were more spread compared with the cells on the BCP scaffold (Fig. 5). This result coincides with the result of previous study. The cells on collagen grafted HA were more spread than those on pure HA [25]. Therefore, it is demonstrated that the collagen in BCP scaffold enhanced the cell attachment ability in early phase.

**Fig. 6:**
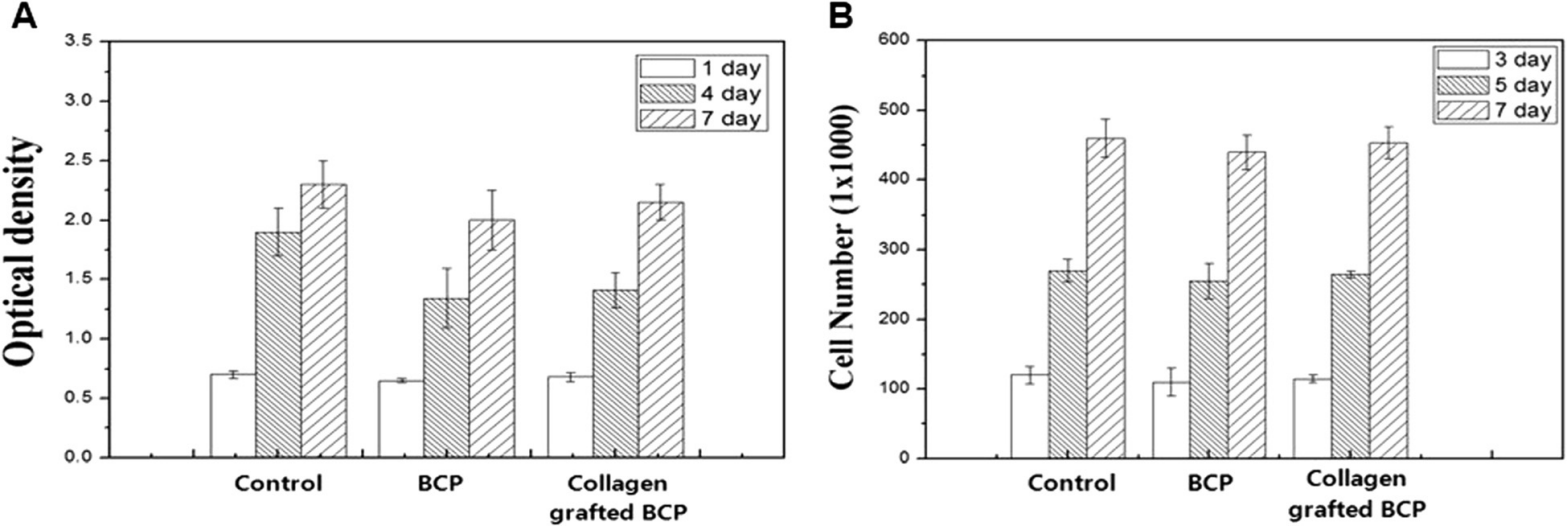
Cell proliferation of MC3T3-E1 cultured on BCP scaffolds and collagen/ BCP scaffolds. Proliferation of MC3T3-E1 cells were determined by MTT assay (**a**) and cell counting (**b**). Data are expressed as the mean ± SD (n = 3)

After incubation for 1, 4, 7 days, the cell proliferation in control, pure BCP and collagen-BCP scaffolds was analyzed by MTT assay. The cell counting in mouse osteoblastic cells (MC3T3-E1 cells) for cultured samples was conducted in terms of incubation periods (3, 5, 7 days). The collagen-BCP scaffold showed similar absorbance and cell number with that of cells on the BCP scaffold for the incubation time (Fig. 6). There was no statistical difference in cell proliferation between the collagen-BCP and pure BCP scaffold (*P* > 0.1).

**Fig. 7:**
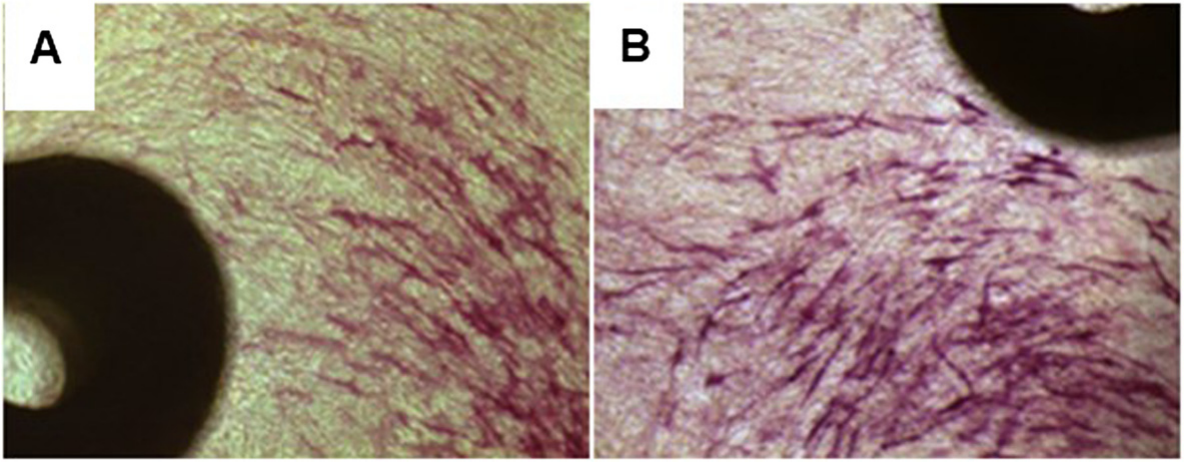
ALP staining of differentiating MC3T3-E1 cultured on BCP scaffolds and collagen/BCP scaffolds. MC3T3-E1 cells were cultured in osteogenic media, and then ALP staining was performed. ALP staining image of BCP scaffolds (**a**) and collagen/BCP scaffolds (**b**)

To evaluate the effects of collagen on osteoblastic differentiation, MC3T3-E1 cells were cultured in osteogenic media, and then ALP staining was performed (Fig. 7). ALP-positive cells on the collagen-BCP scaffold were increased compared with the cells on the pure BCP scaffold. ALP positive cells are shown in red. Even exogenous type I collagen facilitated osteogenic differentiation and acts as a substrate for mineralization [18].

It is believed that the collagen in BCP scaffold enhanced the cell attachment ability in early phase and osteoblastic differentiation. That is, collagen which is bone extracelluar matrix protein may play a critical role in osteoblastic differentiation and phenotypic expression.

## Conclusions

BCP scaffolds were HA/β-TCP phase ratio of 60/40 and had porous microstructure with submicron-sized grains. The collagen was successfully crosslinked into the BCP scaffolds by the EDC/NHS crosslinking method. The cell proliferation of collagen-BCP scaffolds showed a similar pattern to those of the BCP scaffolds. However, cell attachment and osteolbastic differentiation were improved in the collagen-BCP scaffolds. The collagen in the collagen-BCP scaffold was effective in osteoblastic differentiation and phenotypic expression. These results indicate that the collagen-BCP scaffolds with interconnected micropore structures is a good candidate as an osteoconductive bone substitute for the repair of bone defects.

### Availability of supporting data

There was no available supporting data.
